# Gender-related outcome difference is related to course of sepsis on mixed ICUs: a prospective, observational clinical study

**DOI:** 10.1186/cc10277

**Published:** 2011-06-21

**Authors:** Irit Nachtigall, Sascha Tafelski, Andreas Rothbart, Lutz Kaufner, Maren Schmidt, Andrey Tamarkin, Maxim Kartachov, Daniela Zebedies, Tanja Trefzer, Klaus-Dieter Wernecke, Claudia Spies

**Affiliations:** 1 Department of Anaesthesiology and Intensive Care, Charité Universitaetsmedizin Berlin, Campus Charité Mitte and Campus Virchow-Klinikum, Augustenburger Platz 1, 13353 Berlin, Germany; 2 Charité Universitaetsmedizin Berlin and SOSTANA GmbH Berlin, Wildensteiner Straße 27 10318 Berlin, Germany

## Abstract

**Introduction**

Impact of gender on severe infections is in highly controversial discussion with natural survival advantage of females described in animal studies but contradictory to those described human data. This study aims to describe the impact of gender on outcome in mixed intensive care units (ICUs) with a special focus on sepsis.

**Methods**

We performed a prospective, observational, clinical trial at Charité University Hospital in Berlin, Germany. Over a period of 180 days, patients were screened, undergoing care in three mainly surgical ICUs. In total, 709 adults were included in the analysis, comprising the *main population *([female] n = 309, [male] n = 400) including 327 as the sepsis subgroup ([female] *n *= 130, [male] n = 197).

**Results**

Basic characteristics differed between genders in terms of age, lifestyle factors, comorbidities, and SOFA-score (Sequential Organ Failure Assessment). Quality and quantity of antibiotic therapy in means of antibiotic-free days, daily antibiotic use, daily costs of antibiotics, time to antibiotics, and guideline adherence did not differ between genders. ICU mortality was comparable in the main population ([female] 10.7% versus [male] 9.0%; *P *= 0.523), but differed significantly in sepsis patients with [female] 23.1% versus [male] 13.7% (*P *= 0.037). This was confirmed in multivariate regression analysis with OR = 1.966 (95% CI, 1.045 to 3.701; *P *= 0.036) for females compared with males.

**Conclusions**

No differences in patients' outcome were noted related to gender aspects in mainly surgical ICUs. However, for patients with sepsis, an increase of mortality is related to the female sex.

## Introduction

The impact of gender on severe infections is in highly controversial discussion. A natural survival advantage for females [1] in data from animal experiments seems to be contradictory to human clinical data on sepsis-related mortality [2-5]. Eachempati *et al. *[5] demonstrated female gender as an independent predictor of increased mortality in patients with documented infection in a surgical intensive care unit (ICU). He emphasised that different genders may need different types of therapy. Depending on the chosen subgroup, Combes *et al. *[3] reported an increased risk for women to die of nosocomial infections.

Different pathomechanisms were addressed to be responsible for these findings, including sex-related gene polymorphisms [2], effects of sex hormones [6], or different intensities of care, with males receiving more invasive procedures [7,8].

One fundamental issue is to make study populations matchable for research projects (for example, for evaluation of new interventions and drugs), but as well to assess the severity of diseases in mixed populations or for benchmarking purposes. Classification systems like PIRO (predisposition, insult/infection, response, organ dysfunction) included gender with the intention of improving the comparability of studies. But although discussion is nearly a decade old, still no agreement is found as to whether female or male gender is a predisposing factor [9]. Le Gall *et al. *[10] created an extended version of the Simplified Acute Physiology Score-II (SAPS-II), including gender, giving male patients a higher score for predicting mortality [10]. The author excluded burned and coronary and cardiac surgery patients; the latter is a population in which women probably do worse [3]. Interestingly, it seems to be highly cohort related whether men or women are more likely to survive. This study aims to describe the impact of gender on outcome of patients on mixed ICUs with a special focus on sepsis patients.

## Materials and methods

### Study design, location, and patients

This prospective, observational, clinical trial was performed during two 90-day data-acquisition periods from January to March 2006 and February to May 2007 at the Charité University Hospital in Berlin, Germany (tertiary medical care center with 3,200 beds). Three mixed ICUs comprising 61 mainly surgical ICU beds under anesthesiologic management were included.

Patients with ARDS (acute respiratory distress syndrome) or neurologic diagnoses as well as patients from different surgical disciplines, including abdominal, gynecologic, cardiac, and neurosurgery or after severe traumata were screened for inclusion. Every consecutive adult (≥18 years) patient with more than 36 hours of ICU treatment admitted to one of the three ICUs was included prospectively into the study. For the purpose of focusing on anti-infective therapy, only patients with at least one day of antibiotic treatment were included in the analysis.

All patients meeting the criteria for sepsis for at least 1 day during the ICU stay were assigned to a sepsis subgroup. This subgroup was examined independently for primary and secondary study aims.

### Data collection and measurement

Data were recorded in daily rounds from medical records, hospital mainframe computer, and patient data-management system (PDMS; Copra System, Sasbachwalden, Germany). Data were collected every day for the preceding 24 hours.

Data on vital signs, laboratory findings, microbiologic and radiologic diagnostics, anti-infective, vasopressor, and steroid agents, ventilation, pulmonary gas exchange, urine output, and fluid balance were taken from the PDMS.

Information regarding alcohol, drug, or nicotine abuse or immunosuppressive status was taken from the patient's data file. The latter was defined for all patients receiving corticosteroids or other immunosuppressive agents, having HIV or leukemia, or after chemotherapy. TISS-28 (Therapeutic Intervention Scoring System-28), SOFA (Sequential Organ Failure Assessment), and SAPS-II (Simplified Acute Physiology Score-II) scoring systems are measured regularly in included ICUs as surrogate markers for disease severity. Infections were screened by using modified definitions of the Centers for Disease Control and Prevention [11] and the guidelines for the management of adults with hospital-acquired, ventilator-associated, and healthcare-associated pneumonia of the American Thoracic Society [12]. Duration of ventilation was defined as the period of mechanical ventilation support during the ICU stay of the patient (intubated or via tracheostomy). ICU stay was defined as number of days a patient remained in the ICU during one hospital stay, including readmissions from regular wards. Sepsis, severe sepsis, and septic shock are defined according to the national and international sepsis guidelines [13-15]. Patients required demonstration of at least two of four signs of systemic inflammatory response syndrome (body temperature, < 36°C or > 38°C; tachycardia, > 90 beats/min; tachypnea, > 20 per minute, or hypocapnia < 32 mm Hg; leukopenia, < 4,000 per milliliter; or leukocytosis, > 12,000 per milliliter or left shift) associated with an infection.

### Outcome parameters

Primary outcome parameter was ICU mortality rate for male and female patients. As secondary outcome measures, quality and quantity of diagnostic efforts and antibiotic therapy were analyzed. Later, evolution of disease severity by using the TISS-28 (Therapeutic Intervention Scoring System) was examined.

SOP (standard operating procedure) adherence was recorded during data collection but was scored afterward by independent experts. Process of assessment of SOP adherence for treatment and diagnostics was previously described in detail [16].

Time analysis was performed as analysis of sepsis onset, based on the previously mentioned definition and first antibiotic treatment after onset. Duration until antibiotic therapy was estimated between these two time points. Quantity of antibiotics was measured as amount of antibiotic agents per day; prices rely on hospital pharmacy lists from December 2005.

### Statistics and ethical review

All study results are expressed as median with 25% to 75% quartiles (25|75), arithmetic mean (mean) ± standard deviation (± STD), or proportions [%] as appropriate, depending on the proof for normality. For statistical analysis, Wilcoxon-Mann-Whitney tests, the Student *t *tests and χ^2 ^tests were performed as appropriate, with a two-tailed *P *value of < 0.05 considered statistically significant.

Evolution of disease-severity scoring system TISS-28 (Therapeutic Intervention Scoring System) over the clinical course was evaluated by using multivariate nonparametric longitudinal data analysis in a two-factorial design (Brunner analysis). For this analysis, patients were included with at least two sequential values, and number of consecutive days for longitudinal analysis was limited by the days, with at least 50% of the study population providing consistent data. This procedure was chosen a priori to reveal consistent conclusions for the whole population and to reduce the possibility of a selection bias. Finally, the longitudinal analysis compared data of the first consecutive 8 days of the ICU stay. Expected mortality and ratio of observed and expected mortality rates are calculated based on the reported initial SAPS II scoring for each subgroup, as described by Le Gall *et al. *[10].

To compare the risk of mortality for both genders, univariate logistic regression analysis was performed separately incorporating different cofactors (age, alcohol abuse, nicotine abuse, drug abuse, immunosuppressive status, admission category, coexisting diagnoses, infection focus, severe sepsis, septic shock, bacteria detected during ICU stay, and surrogate markers for quality of care). For the purpose of affirming the significant effects of gender on mortality, study results were analyzed by using multivariate logistic regression methods. Based on the results of univariate logistic regression analyses, factors with significant association with mortality were incorporated into multivariate analysis. Multivariate regression analyses also included stepwise backward selection to reveal the most relevant associated parameters for mortality to validate the findings. Odds ratios (ORs) with 95% confidence intervals (CIs) and the corresponding *P *values were calculated for each risk factor. Quality of regression models was assessed with Hosmer-Lemeshow tests for model calibration. All numeric calculations were performed with PASW 18 (SPSS, Inc., Chicago, IL 60606, U.S.A.) and SAS version 9.1 (SAS Institute Inc., 2003, Cary, IN, U.S.A.).

The local Ethics Review Board and the data safety authorities approved this study. The Ethics review board waived the need for patient informed consent to be obtained because of the observational character of the study.

## Results

### Basic characteristics

**Figure 1:**
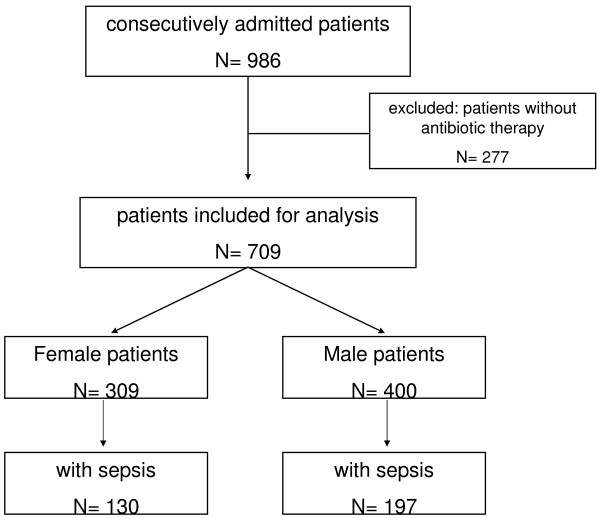
**Flow chart for study enrollment and patient selection**.

Altogether, 986 patients were screened, and 709 with antibiotic treatment were included in further analyses (Figure 1). This main population included 309 women and 400 men, with 130 women and 197 men with sepsis comprising the sepsis subgroup for further analysis.

In the main population, basic characteristics differed between genders in means of age, drug abuse, nicotine abuse, alcohol abuse, vascular diseases, and median SOFA Score on admission. Duration of mechanical ventilation for patients with ventilation support was shorter for women than for men, as displayed in Table 1. Other parameters, like length of ICU stay, immunosuppressive status, comorbidities, admission categories, surgical category, SAPS-II, and TISS-28 on admission did not differ between genders.

**Table 1:** Distribution of basic characteristics between gender in the main population and in the sepsis subgroup

	Main population		Sepsis population	
	♂	♀	♂	♀
	*n *= 400	*n *= 309	*n *= 197	*n *= 130
Age (years)	66 (51|72)^a^	68 (54|78)^a^	64 (50|72)^a^	68 (57|78)^a^
Drug abuse	18 (4.5%)^a^	3 (1.0%)^a^	14 (7.1)^a^	1 (0.8)^a^
Nicotine abuse	56 (14.0%)^a^	15 (4.9%)^a^	25 (12.7)	9 (6.9)
Alcohol abuse	55 (13.8%)^a^	10 (3.2%)^a^	33 (16.8)^a^	8 (6.2)^a^
Preexisting comorbidity				
Immunosuppressive status	27 (6.8%)	31 (10.0%)	11 (5.6%)^a^	18 (13.8%)^a^
Vascular disease	202 (50.5%)^a^	111 (35.9%)^a^	100 (50.8%)^a^	45 (34.6%)^a^
Hypertension	178 (44.5%)	147 (47.6%)	82 (41.6%)	60 (46.2%)
Chronic liver disease	32 (8.0%)	16 (5.2%)	17 (8.6%)	8 (6.2%)
Chronic renal disease	67 (16.8%)	46 (14.9%)	37 (18.8%)	24 (18.5%)
Metabolic disease	172 (43.0%)	141 (45.6%)	102 (51.8%)	71 (54.6%)
Chronic lung disease	45 (11.3%)	37 (12.0%)	27 (13.7%)	20 (15.4%)
Psychiatric disease	81 (20.3%)	47 (15.2%)	53 (26.9%)	31 (23.8%)
Admission category				
Infection	14 (3.5%)	10 (3.2%)	10 (5.1%)	7 (5.4%)
Malignant tumor	65 (16.3%)	80 (25.9%)	27 (13.7%)	21 (16.2%)
Neurology	43 (10.8%)	34 (11.0%)	23 (11.7%)	20 (15.4%)
Cardiovascular	154 (38.5%)	90 (29.1%)	67 (34.0%)	34 (26.2%)
Respiratory	21 (5.3%)	17 (5.5%)	15 (7.6%)	16 (12.3%)
Gastrointestinal	30 (7.5%)	19 (6.1%)	20 (10.2%)	9 (6.9%)
Trauma	42 (10.5%)	29 (9.4%)	23 (11.7%)	11 (8.5%)
Others	31 (7.8%)	30 (9.7%)	12 (6.1%)	12 (9.2%)
Surgical patients	327 (81.8%)	262 (84.8%)	151 (76.6%)	100 (76.9%)
OP category of surgical patients				
Neurosurgical	38 (11.6%)	40 (15.3%)	19 (12.6%)	19 (19.0%)
Musculoskeletal	59 (18.0%)	46 (17.6%)	33 (21.9%)	19 (19.0%)
Cardiac	134 (41.0%)	65 (24.8%)	52 (34.4%)	20 (20.0%)
Abdomen and urogenital	83 (25.4%)	97 (37.0%)	41 (27.2%)	30 (30.0%)
Thorax	9 (2.8%)	9 (3.4%)	4 (2.6%)	7 (7.0%)
Soft tissue and others	4 (1.2%)	5 (1.9%)	2 (1.3%)	5 (5.0%)
Severity of disease scorings				
SAPS-II on admission	35 (26|47)	34 (26|45)	39 (28|51)	40 (29|53)
SOFA on admission	5 (2|8)^a^	4 (2|7)^a^	6 (4|9)^a^	5 (3|7)^a^
TISS-28 on admission	35 (26|42)	33 (27|40)	37 (29|44)	35 (28|41)
Diagnostic patterns				
Microbiologic diagnostics (% of LOS)	17.0 (± 20.4)	14.9 (± 19.8)	27.6 (± 19.3)	24.3 (± 17.3)
Radiologic diagnostics of (% of LOS)	55.2 (± 30.0)^a^	51.1 (± 31.0)^a^	52.1 (± 23.0)^a^	45.4 (± 21.3)^a^
Antibiotic therapy				
SOP adherence (% of LOS)	77.3 (± 32.6)	79.0 (± 33.2)	68.9 (± 34.4)	69.3 (± 36.4)
DAU in agents per day	1.0 (0.5|1.5)	1.0 (0.5|1.5)	1.26 (± 0.65)	1.18 (± 0.74)
Antibiotic-free days (% of LOS)	28.8 (± 27.9)	27.3 (± 27.6)	22.0 (± 22.4)	25.4 (± 24.3)
Daily costs for antibiotics in €	19.3 (± 25.5)	15.6 (± 21.6)	30.7 (± 28.6)	26.7 (± 27.1)
Duration of treatment				
ICU stay in days	4 (2|11)	4 (2|9.5)	10 (5|19)	10 (5|20)
Invasive ventilation in hours^b^	27 (11|121)^a^	20 (8|95)^a^	96 (22|305)	85 (16|300)

^a^*P *< 0.05 in two-tailed significance tests. ^b^Ventilated patients in main population, *n *= ♂ 331, ♀ 226; in sepsis subgroup, *n *= ♂ 182, ♀ 115. Binary parameters given in total and percentage (%), continuous variables presented in median and 25% and 75% quartiles (25%|75%) or mean (± STD). DAU, daily antibiotic use; LOS, length of stay; OP, operation; SAPS, simplified acute physiology score-II; SOFA, sequential organ-failure assessment; SOP, standard operating procedure; TISS-28, therapeutic intervention scoring system-28.

Subgroup analysis for sepsis patients showed differences of basic characteristics in age, drug abuse, alcohol abuse, immunosuppressive status, vascular disease, and median SOFA Score on admission, as summarized in Table 1. Other parameters like duration of mechanical ventilation, length of ICU stay, comorbidities, admission categories, surgical category, SAPS-II, and TISS-28 on admission did not differ between genders.

### Diagnostic efforts, patterns of infections, and antibiotic therapy

In the main population, microbiologic diagnostics in relation to length of ICU stay (% LOS) did not differ, but radiologic diagnostics was reduced for women (Table 1). A higher percentage of men had an infection, and pneumonia was less often seen in women, but conversely, lower urinary tract infections were more common in women (Table 2). Notably, quality of antibiotic therapy in means of antibiotic-free days, daily antibiotic use (DAU) in agents per day, daily costs of antibiotics, and SOP adherence in percentage of all ICU days did not differ between genders (Table 1).

**Table 2:** Distribution of infections, infection characteristics, and pathogens for main and sepsis population

	Main population		Sepsis population	
	♂	♀	♂	♀
Number	400	309	197	130
Patients with infections *n *(%)	248 (62.0%)^a^	164 (53.1%)^a^	197 (100%)	130 (100%)
Colitis, pseudomembranous	2 (0.5%)	3 (1.0%)	2 (1.0%)	2 (1.5%)
Pneumonia	135 (33.8%)^a^	75 (24.3%)^a^	124 (62.9%)	70 (53.8%)
Lower urinary tract infections	13 (3.3%)^a^	34 (11.0%)^a^	11 (5.6%)^a^	30 (23.1%)^a^
Bones and joints	11 (2.8%)	7 (2.3%)	8 (4.1%)	6 (4.6%)
Endocarditis	11 (2.8%)	7 (2.3%)	10 (5.1%)	6 (4.6%)
Abdomen	40 (10.0%)	28 (9.1%)	32 (16.2%)	18 (13.8%)
Soft tissue and wounds	80 (20.0%)	47 (15.2%)	55 (27.9%)	38 (29.2%)
Upper urinary tract infections	6 (1.5%)	4 (1.3%)	3 (1.5%)	1 (0.8%)
Meningitis	5 (1.3%)	5 (1.6%)	4 (2.0%)	3 (2.3%)
Bloodstream infection	35 (8.8%)	17 (5.5)%)	34 (17.3%)	16 (12.3%)
Fever of unknown origin	33 (8.3%)	24 (7.8%)	22 (11.2%)	17 (13.1%)
Catheter-related infections	17 (4.3%)	5 (1.6%)	17 (8.6%)	5 (3.8%)
SIRS occurrence *n *(%)	364 (91.0%)	290 (93.9%)	197 (100%)	130 (100%)
Sepsis occurrence *n *(%)	197 (49.3%)	130 (42.1%)	197 (100%)	130 (100%)
Severe sepsis occurrence *n *(%)	89 (22.3%)^a^	48 (15.5%)^a^	89 (45.2%)	48 (36.9%)
Septic shock occurrence *n *(%)	74 (18,5%)	41 (13,3%)	74 (37.6%)	41 (31.5%)
Fungi detected	64 (16.0%)	47 (15.2%)	61 (31.0%)	42 (32.3%)
Gram-negative germs detected	98 (24.5%)	58 (18.8%)	89 (45.2%)	52 (40.0%)
Difficult-to-treat bacteria detected	41 (10.3%)	28 (9.1%)	37 (18.8%)	26 (20.0%)

^a^*P *< 0.05 in a two-tailed χ^2 ^test. More than one infection period per patient was possible. Difficult-to-treat pathogens include bacteria associated with potential intrinsic or acquired resistance (methicillin-resistant *Staphylococcus aureus*, germs producing extended spectrum betalactamases, *Vancomycin*-resistant Enterococci, nonfermenters like *Pseudomonas *spp., *Stenotrophomonas maltophilia, Acinetobacter baumanii, Citrobacter *spp., *Enterobacter cloacae, Enterococcus faecium*, and *Bacillus cereus*). Binary parameters presented in total and percentage (%). SIRS, systemic inflammatory response syndrome.

Similarly, in the sepsis subgroup, radiologic diagnostics was performed less often in women (Table 1). Distribution of urinary tract infections differed significantly between males and females (Table 2). The remaining parameters were equally distributed between groups (Tables 1 and 2).

### Time to antibiotic therapy

For the sepsis subgroup, no statistically significant difference appeared for the time to antibiotics. Duration from onset of sepsis to antibiotic therapy in median was ♀ 0.54 h (25%|75% Quartiles 0.0|4.70 h) versus ♂ 1.5 h (25%|75% quartiles, 0.0|6.25 h; *P *= 0.126).

### Mortality

**Figure 2:**
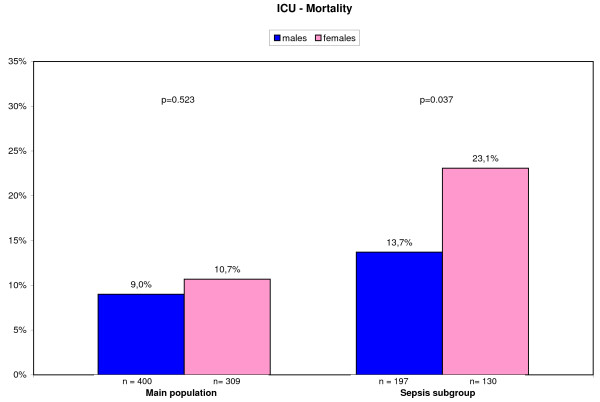
**ICU mortality for main study population and sepsis subgroup**.

ICU mortality in the main study population was equal between both genders (♀ 10.7% versus ♂ 9.0%; *P *= 0.523), but differed significantly in the sepsis subgroup (♀ 23.1% versus ♂ 13.7%; *P *= 0.037), as displayed in Figure 2.

The O/E mortality rate in the main population was 0.539 (95% CI, 0.378 to 0.747) for men and 0.699 (95% CI, 0.481 to 0.981) for women; in the sepsis population 0.596 (95% CI, 0.393 to 0.869) for men and 0.935 (95% CI, 0.629 to 1.332) for women, respectively [10].

In the female population, in 2.9%, therapy was discontinued. In male patients, this proportion was nearly equally contributed with 2.3% of patients, *P *= 0.634.

### Logistic regression analysis

With univariate logistic regression, risk factors for mortality were assessed (Table 3). For the main study, population age, TISS-28 on admission, occurrence of infection during ICU stay, pneumonia, and septic shock were significantly associated with mortality. Alternatively to TISS-28, severity of disease scoring system SAPS-II also showed this association, with an OR of 1.066 (95% CI, 1.048 to 1.085). Additionally, difficult-to-treat pathogens and SOP adherence < 65% were also significantly associated with mortality.

**Table 3:** Effects of different factors on outcome in a mixed ICU population for study population

	Univariate logistic analysis		Multivariate logistic analysis	
Parameter	OR (95% CI)	*P*	OR (95% CI)	*P*
Gender ♀ vs. ♂	1.209 (0.735-1.988)	0.455	1.277 (0.720-2.264)	0.403
Age	1.035 (1.015-1.055)	< 0.001	1.041 (1.018-1.064)	< 0.001
Alcohol abuse	1.341 (0.611-2.943)	0.464		
Nicotine abuse	0.526 (0.186-1.490)	0.227		
Drug abuse	0.976 (0.222-4.280)	0.974		
Immune suppression	0.668 (0.234-1.904)	0.450		
TISS-28 first	1.080 (1.053-1.108)	< 0.001	1.066 (1.035-1.097)	< 0.001
Infection during ICU stay	3.881 (1.745-8.633)	0.001	1.269 (0.487-3.304)	0.626
				
Infection type				
Pneumonia	4.370 (2.613-7.311)	< 0.001	1.377 (0.676-2.802)	0.378
Urinary tract infection	1.694 (0.728-3.941)	0.221		
Abdomen	1.477 (0.698-3.127)	0.308		
Bones and joints	1.164 (0.262-5.173)	0.842		
Endocarditis	1.164 (0.262-5.173)	0.842		
Meningitis	1.031 (0.129-8.262)	0.977		
Wounds/soft issue	1.310 (0.713-2.404)	0.384		
BSI	2.338 (0.258-21.220)	0.450		
Colitis, pseudomembranous	2.338 (0.258-21.220)	0.450		
Infection of unknown origin	0.883 (0.341-2.292)	0.799		
Severe sepsis^a^	4.110 (2.448-6.900)	< 0.001		
Septic shock	5.023 (2.963-8.515)	< 0.001	2.746 (1.328-5.680)	0.006
ICU diagnoses				
Infection	2.553 (0.922-7.068)	0.071		
Neoplastic	0.556 (0.269-1.149)	0.113		
Neurologic	0.348 (0.107-1.134)	0.08		
Cardiovascular	1.170 (0.700-1.956)	0.548		
Respiratory	4.306 (2.032-9.125)	< 0.001	1.447 (0.542-3.860)	0.461
Gastrointestinal	1.322 (0.542-3.229)	0.540		
Trauma	0.526 (0.186-1.490)	0.227		
Miscellaneous	0.815 (0.315-2.108)	0.673		
Postoperative admission	0.865 (0.457-1.637)	0.655		
Type of surgery				
Head	0.905 (0.399-2.053)	0.811		
Musculoskeletal	0.972 (0.481-1.967)	0.938		
Cardiac	1.136 (0.661-1.951)	0.645		
Abdomen/urogenital	0.658 (0.351-1.233)	0.191		
Thorax	1.164 (0.262-5.173)	0.842		
Soft tissues and peripheral vascular	4.803 (1.174-19.651)	0.029	3.509 (0.613-20.073)	0.158
Fungi detected	4.255 (2.492-7.266)	< 0.001	1.398 (0.701-2.788)	0.341
Gram-negative bacteria detected	2.929 (1.748-4.906)	< 0.001	1.234 (0.605-2.513)	0.563
Difficult-to-treat pathogens detected^b^	2.421 (1.248-4.697)	0.009	1.115 (0.499-2.493)	0.791
SOP adherence ≤65%	1.705 (1.011-2.874)	0.045	2.309 (1.182-4.511)	0.014

Effects of different factors on outcome in a mixed ICU population for study population (*N *= 709) by using univariate logistic regression analyses. Parameters found to be relevant on a *P *< 0.05 level for ICU mortality entered multivariate logistic regression analysis. Quality of regression model tested with the Hosmer-Lemeshow test, indicating good calibration (χ^2 ^= 4.672; *P *= 0.792). BSI, bloodstream infection; SOP, standard operating procedure; TISS-28, therapeutic intervention scoring system-28.

^a^Septic shock entered multivariate analysis due to correlation; ^b^Difficult-to-treat pathogens include bacteria associated with potential intrinsic or acquired resistance (methicillin-resistant *Staphylococcus aureus*, germs producing extended spectrum betalactamases, vancomycin-resistant Enterococci, nonfermenters like *Pseudomonas *spp., *Stenotrophomonas maltophilia, Acinetobacter baumanii, Citrobacter *spp., *Enterobacter cloacae, Enterococcus faecium*, and *Bacillus cereus*).

Based on these findings, a multivariate logistic regression model was performed to affirm the effect of gender adjusted to other relevant factors for mortality. The resulting odds ratio for the parameter gender was 1.277 for women compared with men but without reaching a level of significance (Table 3).

The same methods were used to evaluate the sepsis subgroup with gender, age, TISS-28 on admission, occurrence of pneumonia, septic shock, and adherence to SOPs < 65% significantly associated with ICU mortality (Table 4). The resulting multivariate logistic regression model showed gender as a significant factor for mortality in this population, with an odds ratio of 1.909 for women compared with men in the full model. A stepwise backward-selection procedure reproduced this finding in a reduced model with four variables (gender, age, TISS-28 on admission, and septic shock), with an OR for mortality of 1.966 for women compared with men (Table 5).

**Table 4:** Effects of different factors on outcome for sepsis subgroup (*n *= 327) by using univariate logistic regression analyses

	Univariate logistic analysis		Multivariate logistic analysis	
Parameter	OR (95% CI)	*P*	OR (95% CI)	*P*
Gender ♀ vs. ♂	1.889 (1.062-3.359)	0.030	1.909 (1.002-3.638)	0.049
Age	1.036 (1.014-1.059)	0.001	1.037 (1.013-1.063)	0.003
Alcohol abuse	0.972 (0.408-2.317)	0.948		
Nicotine abuse	0.428 (0.126-1.453)	0.174		
Drug abuse	0.719 (0.158-3.277)	0.670		
Immune suppression	0.740 (0.247-2.214)	0.590		
TISS-28 first	1.063 (1.033-1.093)	< 0.001	1.062 (1.028-1.098)	< 0.001
Infection type				
Pneumonia	1.960 (1.048-3.664)	0.035	1.120 (0.539-2.324)	0.762
Urinary tract infection	0.790 (0.316-1.977)	0.614		
Abdomen	1.047 (0.477-2.298)	0.908		
Bones and joints	0.782 (0.170-3.593)	0.752		
Endocarditis	0.665 (0.147-3.010)	0.596		
Meningitis	0.786 (0.093-6.655)	0.825		
Wounds/Soft issue	0.787 (0.408-1.519)	0.476		
BSI	1.589 (0.162-15.560)	0.691		
Colitis, pseudomembranous	1.589 (0.162-15.560)	0.691		
Infection of unknown origin	0.667 (0.249-1.788)	0.421		
Severe sepsis^a^	1.845 (1.037-3.280)	0.037		
Septic shock	2.418 (1.354-4.319)	0.003	1.649 (0.854-3.184)	0.136
ICU diagnoses				
Infection	1.492 (0.468-4.755)	0.499		
Neoplastic	0.939 (0.414-2.130)	0.880		
Neurologic	0.319 (0.095-1.071)	0.065		
Cardiovascular	1.146 (0.624-2.107)	0.660		
Respiratory	2.523 (1.117-5.699)	0.026	1.374 (0.521-3.620)	0.520
Gastrointestinal	1.263 (0.490-3.259)	0.629		
Trauma	0.428 (0.126-1.453)	0.174		
Miscellaneous	0.943 (0.310-2.873)	0.918		
Postoperative admission	1.165 (0.581-2.336)	0.667		
Type of surgery				
Head	1.079 (0.450-2.589)	0.864		
Musculoskeletal	0.573 (0.232-1.414)	0.227		
Cardiac	1.335 (0.691-2.577)	0.390		
Abdomen/Urogenital	0.954 (0.474-1.919)	0.894		
Thorax	1.055 (0.222-5.016)	0.947		
Soft tissues and peripheral vascular	3.694 (0.804-16.981)	0.093		
Fungi detected	2.297 (1.282-4.118)	0.005	1.523 (0.782-2.968)	0.216
Gram-negative bacteria detected	1.341 (0.756-2.379)	0.315		
Difficult-to-treat pathogens detected^b^	1.300 (0.652-2.594)	0.457		
SOP adherence ≤65%	1.139 (0.632-2.052)	0.664		

Parameters found to be relevant on a *P *< 0.05 level for ICU mortality entered multivariate logistic regression analysis. Quality of regression model tested with Hosmer-Lemeshow test indicating good calibration (χ^2 ^= 7.014; *P *= 0.535). BSI, blood stream infection; SOP, standard operating procedure.

^a^Septic shock entered multivariate analysis due to correlation; ^b^Difficult-to-treat pathogens include bacteria associated with potential intrinsic or acquired resistance (methicillin-resistant *Staphylococcus aureus*, germs producing extended spectrum betalactamases, vancomycin-resistant Enterococci, Nonfermenters like *Pseudomonas *spp., *Stenotrophomonas maltophilia*, *Acinetobacter baumanii*, Citrobacter spp., *Enterobacter cloacae*, *Enterococcus faecium*, and *Bacillus cereus*).

**Table 5:** Multivariate logistic regression analysis in a stepwise backward model

	Multivariate logistic analysis	
Parameter	OR (95% CI)	*P*
Gender ♀ vs. ♂	1.966 (1.045-3.701)	0.036
Age	1.038 (1.013-1.063)	0.002
TISS-28 first	1.069 (1.036-1.103)	< 0.001
Septic shock	1.831 (0.977-3.432)	0.059

Multivariate logistic regression analysis in a stepwise backward model confirming associations with mortality shown in Table 4: Hosmer-Lemeshow test for the last step indicating good calibration (χ^2 ^= 7.402; *P *= 0.494). TISS-28, therapeutic intervention scoring system-28.

### Analysis of the progressive course of scoring system TISS-28

**Figure 3:**
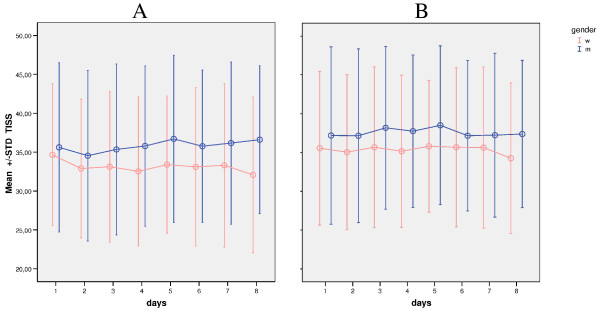
**TISS-28 scoring for severity of disease comparing gender over the first 8 consecutive ICU days**. Error bars illustrate the evolution of TISS-28 (Therapeutic Intervention Scoring System-28) scoring for severity of disease comparing gender (m, men; w, women) over the first 8 consecutive ICU days. STD, standard deviation. **(a) **In the main population, differences between groups were statistically significant for Gender (*P *< 0.001) and for interaction between Gender and Time (*P *= 0.029), but not for Time (*P *= 0.063). **(b) **In the sepsis subgroup, differences between groups were statistically significant for Gender (*P *= 0.018) but not for interaction between Gender and Time (*P *= 0.662) or Time (*P *= 0.257).

Evolution of TISS-28 scoring for severity of disease for men and women was compared via Brunner analysis over the first eight consecutive ICU days. Differences between groups were statistically significant in the nonparametric multivariate analysis for longitudinal data for the first independent factor, Gender (*P *< 0.001), and for interaction between Gender and Time (*P *= 0.029) but not for the second dependent factor, Time (*P *= 0.063). In the sepsis subgroup, differences between groups were statistically significant for Gender (*P *= 0.018) but not for Time (*P *= 0.257) and for the interaction between Gender and Time (*P *= 0.662). These findings are also reflected in Figure 3, illustrating the different development of TISS-28 over time for women and men despite initially similar levels.

## Discussion

Most important, we were able to demonstrate that a gender-related effect on mortality is limited to the specific subgroup of sepsis patients. Further, we showed that despite smaller differences in the care process, quality and quantity of key interventions in infection management are distributed equally between genders. These findings contribute to the discussion of whether different levels of care have an impact on gender-related outcome [8,17,18].

Concerning distribution of basic characteristics, we were able to reproduce well-described differences between genders comparing lifestyle risks, comorbidities, and age [3,8]. Similarly, subgroup analysis for sepsis patients showed differences in basic characteristics for age, lifestyle risks, immunosuppressive status, and vascular disease. Women had lower SOFA score on admission in the main study population as well as in the sepsis subgroup. This finding is well described for other study populations [19]. However, primary assessed scores reflect only the moment of admission but not the kinetic of clinical course, for which SOFA is not validated [20]. Because this scoring system does not take gender into account [21,22], the gender-related difference in mortality is not reflected well and remains a topic that must be elucidated.

Quantity of microbiologic diagnostics did not differ between genders, but in men, more radiologic diagnostics were performed. This is consistent with the findings of Valentin *et al. *[8], that men are more likely to obtain a higher intensity of care in ICU. Furthermore in our study, no differences were found for antibiotic-free days, daily antibiotic use, daily costs of antibiotics, and SOP adherence in percentage of all days--neither in the main study population nor in the sepsis subgroup--showing that in our study population, men and women get the same quality and quantity of care in means of antibiotic therapy. As time to antibiotics was found to be relevant for ICU mortality, we also included this parameter in our analysis but did not find significant differences between genders [23].

In women, pneumonia as well as overall infection rate was lower, but urinary tract infections occurred more often. This goes along well with previously described data [3,4,24,25]. A higher infection rate in men, mainly based on increased pneumonia rates, may impair survival. Conversely, in our main population, outcome of gender was equal, but in the sepsis subgroup, women were more likely to die than were men. As we think that gender differences are a matter of subgroup selection, this finding is in concordance with other published studies in mixed ICUs, where several differences for gender, but not for mortality, are described [3]. Experimental data show a natural survival advantage after polymicrobial sepsis for women [1], but human studies focusing the impact of gender on sepsis-related mortality gave inconsistent results, showing a lower [7,19], equal [26,27] or higher [5,28-30] mortality rate. Vincent *et al. *[29] reported, in their European multicenter cohort, higher mortality for women with sepsis than for men [29]. Combes *et al. *[3], studying ICU patients with sepsis, reported that female gender predicted mortality, although in the univariate analysis, mortality was not significantly different. Seymour *et al. *[28] reported a higher mortality rate for women with Fournier gangrene, with more male patients surviving the septic phase. In contrast to this, Adrie *et al. *[29] found a survival advantage in women older than 50 years, which is also opposed to our findings [19]. In that study, Adrie *et al. *postulated women older than 50 years to be postmenopausal without measuring hormone status, which seems to be of high importance. Further on, they included only community-acquired sepsis and predominantly medical patients, which is a difference from our and other study populations. The results of the O/E mortality rate suggest that the SAPS-II score provides the most reliable predictive values for the subgroup of women with sepsis. It is an interesting finding that might indicate a limitation of intensive care scoring systems.

In our study, we also analyzed evolution of TISS-28 scoring for men and women. We found consistent differences between genders being statistically significant. As TISS-28 is a surrogate parameter for intensity of care, our results coincide with the findings that men receive more invasive procedures than do women, regardless of the reason of admission or procedures [7,8]. Conversely, TISS-28 was thought to be used to compare predicted survival in different populations [21,22]. Based on these findings, it might be difficult to use scoring systems not incorporating gender as an independent factor for ICU mortality.

## Limitations

Our study has several limitations. First, data were collected in one tertiary care center but with three ICUs with a very mixed population.

Second, we did not measure sex-hormone levels and did not stratify for age, because postmenopausal status is difficult to assess without measuring sex hormones. As experimental data showed an influence of sex hormones on outcome, this might alter our results. These data advocate further prospective trials with specific focus on this issue.

Third, we did not take into account marital status, which is meant to have an influence on mortality from sepsis [28]. Although having probably the same impact on both genders, the fact that data were collected only during winter and spring could theoretically represent another limitation.

Fourth, 81.8% of our patients are surgical, so results for medical patients might be different.

Finally, studies of gender-related differences are limited to nonrandomized designs. As in studies of other not-modifiable factors like hospital admission time, ethnicity, or genotype we cannot rule out the possibility of further confounders or interactions that were not addressable in our careful multivariate evaluation [31].

## Conclusions

In our population, it seems as if only in a sepsis subgroup gender makes a difference and that if women develop sepsis, they do worse than men. Thus, as sepsis occurred more frequently in male patients, prevention measures for infections might be more important for men, but we should pay more attention to specific therapies for women, because of their higher attributable mortality if seriously infected.

## Key messages

• Gender-related effect on mortality is limited to the specific subgroup of sepsis patients.

• Quality and quantity of key interventions in infection management are distributed equally between genders.

• ICU scoring systems should take gender into account as an independent factor for mortality.

## Abbreviations

ARDS: acute respiratory distress syndrome; BSI: bloodstream infection; DAU: daily antibiotic use; ESBL: extended spectrum betalactamase; ICU: intensive care unit; LOS: length of stay; MRSA: methicillin-resistant *Staphylococcus aureus*; OP: operation; PDMS: patient data-management system; PIRO: predisposition, insult/infection, response, organ dysfunction; SAPS-II: simplified acute physiology score-II; SIRS: systemic inflammatory response syndrome; SOFA: sequential organ-failure assessment; SOP: standard operating procedure; STD: standard deviation; TISS-28: therapeutic intervention scoring system-28; VRE: vancomycin-resistant enterococcus.

## Competing interests

IN and ST received funding from Roche GmbH. AT received funding from SIRS-Lab GmbH Jena. CS was funded by Merck Sharp and Dohme GmbH, Astra Zeneca, Bristol-Myers Squibb GmbH, Pfizer, and Fresenius Kabi. KDW, DZ, MS, MK, LK, and AR declare that they have no competing interests. No involvements existed in study design, in the collection, analysis, and interpretation of data, in the writing of the report, or in the decision to submit the paper for publication.

## Authors' contributions

IN, ST, DZ, MK, and CS worked out the design and conception of the study and were responsible for data acquisition, analyses, and interpretation. IN, ST, LK, and MS revised the primary study data. ST and KDW performed statistical analyses and prepared the presentation. IN and ST drafted the final manuscript in equal parts. TT, AR, and AT revised all presented data and reevaluated the manuscript. The corresponding author had full access to all the data in the study and had final responsibility for the decision to submit for publication. All authors read and approved the manuscript for publication.
